# Current research and management of undifferentiated pleomorphic sarcoma/myofibrosarcoma

**DOI:** 10.3389/fgene.2023.1109491

**Published:** 2023-02-16

**Authors:** Haitao Sun, Jilu Liu, Fangyuan Hu, Meng Xu, Ao Leng, Feng Jiang, Kefu Chen

**Affiliations:** ^1^ Department of Spine Surgery, Naval Hospital of Eastern Theater Command, Zhoushan, China; ^2^ Department of Orthopaedics, General Hospital of Northern Theater Command, Shenyang, China; ^3^ Department of Neonatology, Obstetrics and Gynecology Hospital of Fudan University, Shanghai, China; ^4^ The No.988th hospital of Joint Logistic Support Force of PLA, Zhengzhou, China; ^5^ Institute of Neuroscience, Key Laboratory of Molecular Neurobiology of Ministry of Education and the Collaborative Innovation Center for Brain Science, Naval Medical University, Shanghai, China

**Keywords:** undifferentiated pleomorphic sarcoma, myxofibrosarcoma, sarcomagenesis, signaling pathways, treatment

## Abstract

Undifferentiated pleomorphic sarcoma (UPS), once termed as malignant fibrous histiocytoma, has always been diagnosed exclusively in clinical practice because it lacks any defined resemblance to normal mesenchymal tissue. Although myxofibrosarcoma (MFS) has been separated from UPS due to its fibroblastic differentiation with myxoid stroma, UPS and MFS are still identified as a sarcoma group in terms of molecular landscapes. In this review article, we will describe the associated genes and signaling pathways involved in the process of sarcoma genesis and make a summary of conventional management, targeted therapy, immunotherapy, and some novel potential treatments of UPS/MFS. With the progressive advancements in medical technology and a better understanding about the pathogenic mechanism of UPS/MFS in the coming decades, new lights will be shed on the successful management of UPS/MFS.

## Introduction

Undifferentiated pleomorphic sarcoma (UPS), once termed as malignant fibrous histiocytoma, is one of the most frequent soft tissue sarcoma (Weiss and Enzinger, 1978; Carneiro et al., 2009). Myxofibrosarcoma (MFS), histologically similar to UPS, was segregated from UPS, and re-classified as an individual entity in 2002 on the ground of its clinic pathology (Hu et al., 2020). However, comprehensive, integrated genomics shows that UPS and MFS are largely indistinguishable across the multi-platform molecular landscape (Cancer Genome Atlas Research Network and Cancer Genome Atlas Research Network, 2017). Therefore, we regard UPS/MFS as a single spectrum of disease herein. UPS/MFS, characterized by highly genetic complexity, has always been the puzzle in clinical practice. Profound knowledge of the pathogenesis of UPS/MFS is missing, and the definite diagnostic characteristics and therapeutic strategies are necessary. For a better understanding of UPS/MFS, advances on the identification of aberrant signaling involved in sarcoma genesis, advancement of conventional management, and development of targeted therapy and immunotherapy, as well as novel therapy with promising future, are required.

## Who opens Pandora's box of UPS/MFS?

The genetically “complex” sarcoma, UPS/MFS, arises due to chromosomal aberrations and/or genetic alterations, but the exact mechanism remains elusive. And the significance of tumor microenvironment is certainly demonstrated in UPS/MFS.

### Presence of special molecules and associated signaling

The oncogenic driver genes and related signaling pathways underlying the sarcoma genesis of UPS/MFS in terms of cell proliferation, invasion, and migration have been widely debated. Mutational profiles and genomic alterations indicate that signaling pathways such as PI3K/Akt/mTOR and Hippo signaling pathways are frequently affected (Cancer Genome Atlas Research Network and Cancer Genome Atlas Research Network, 2017; Zheng et al., 2019; Ye et al., 2020; Ali et al., 2019; Rivera-Reyes et al., 2018), because of genomic, transcriptional, and proteomic alterations in UPS/MFS (Cancer Genome Atlas Research Network and Cancer Genome Atlas Research Network, 2017; Ali et al., 2019; Zhou et al., 2018; Romeo et al., 2012) (Shown in Table 1)

**TABLE 1:** The molecular landmark and potential pathways for sarcomagenesis of UPS/MFS.

Targeting molecule	Genomic profiling	Transcriptomic profiling	Morphoproteomic profiling
*TP53/ARF/MDM2 pathway*			
TP53	subs, trun, homD, hetD	—	—
ING1	homD, hetD, amp	—	—
MDM2	HLA, hetD	—	—
CDKN2A/CDKN2B (INK4A/ARF/INK4B)	hypermethylation, hmD, hetD	—	—
miR-223	—	downregulation	—
Skp2	—	—	overexpression
*RB1/CDK4/INK4A/INK4B pathway*			
CDKN2A/CDKN2B (INK4A/ARF/INK4B)	homD, hetD, hypermethylation	—	—
RB1	HLA, mut, homD, hetD	—	—
CDK4	hmoD, amp	—	—
*Chromatin remodelling pathway*			
ATRX	mut, amp, homD	—	—
H3F3A	mut	—	—
DOT1L	mut	—	—
*RAS/MAPK pathway*			
KRAS	mut	—	—
DICER	haloinsufficiency	—	—
Braf	mut	—	—
MET	HLA	—	—
NF1	trun, subs	—	—
MAP2K1	gain	—	—
PRKCA	—	—	overexpression
MAPK1	—	—	overexpression
FGFR2	amp	—	—
*PI3K/Akt/mTOR pathway*			
PIK3CA	mut	—	—
TSC1	trun	—	—
RICTOR	amp	—	—
STK11	rearrangement	—	—
PTEN	del	—	—
ITGA10	hypomethylation	upregulation	—
PPP2R2B	methylation	downregulation	—
HSP90	—	—	overexpression
NF1	trun, subs	—	—
MET	—	—	overexpression
KIT	gain, loss	—	overexpression
HIF-2α	—	downregulation	—
miR-152	—	upregulation	—
IGF1R	—	—	overexpression
Akt2	mut	—	phosphorylation
mTOR	—	—	phosphorylation
4BEP	—	—	phosphorylation
FGFR2	amp	—	—
*Hedgehog and notch pathway*			
GLI1	—	upregulation	—
GLI2	—	—	overexpression
PTCH1	—	upregulation	—
HHIP	—	upregulation	—
HES1	—	upregulation	—
HEY1	—	upregulation	—
HEY2	—	upregulation	—
miR-199b-5p	—	upregulation	—
*Cell cycle pathway*			
CDK6	amp	—	—
CDK8	amp	—	—
CCNE1	HLA	—	—
CCND1	amp	—	—
*Hippo pathway*			
YAP	CNV	—	overexpression
TAZ (WWTR1)	—	—	overexpression
VGLL3	CNV	—	—
*NF-κB pathway*			
YAP	CNV	—	overexpression
*NF-κB*	—	—	phosphorylation
*TGFβ–YAP1–RHAMM pathway*			
HMMR/RHAMM		upregulation	overexpression
TGFβR1	amp	upregulation	—
TGFβ1	amp	upregulation	—
SMAD3	fusion, amp	upregulation	—
SMAD4	mut	—	—
FXOM1	amp	upregulation	—
ATRX	amp, trun	upregulation	—
*Hypoxia-related pathway*			
STAT1	—	—	overexpression
HIF-1α	—	upregulation	overexpression
PLOD2	—	—	overexpression
*The urokinase-type plasminogen activation system*			
UPA/PLAU	amp	upregulation	overexpression
*JAK-STAT pathway*			
STAT3	—	—	dephosphorylation
JAK2	—	—	overexpression
*Wnt2/β-catenin pathway*			
Dkk1	—	—	overexpression
*CLIC1 signaling pathway*			
CLIC1	—	—	overexpression
*Anchorage-independent growth, pro-inflammation pathway*			
TRIO-TERT	gene fusion	—	—
COX-2	—	—	overexpression
*Epithelial mesenchymal transition*			
Snail	—	downregulation	—
MMP2	—	upregulation	—
MMP9	—	downregulation	—

Abbreviations: amp, amplification; CNV, copy number variation; del, Deletion; HLA, high-level amplification; homD, homozygous deletion; hetD, heterozygous deletion; mut, mutation; subs, Substitution; trun, Truncation.

**FIGURE 1:**
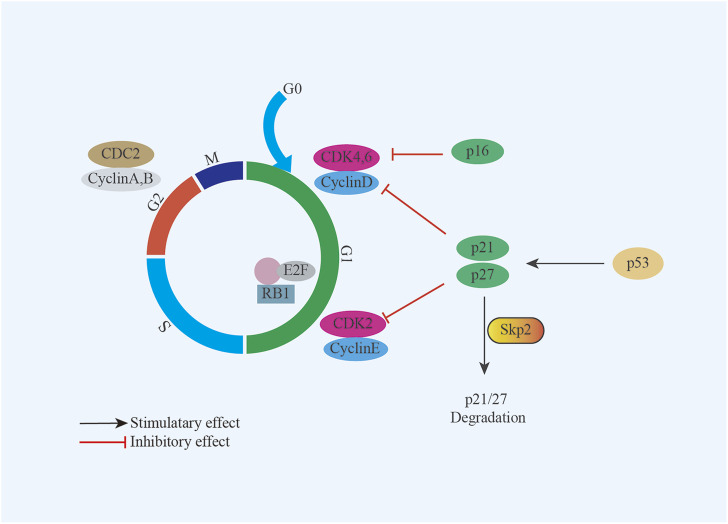
Model summarizing the role of Skp2 and p16 in UPS/MFS. TP53 deficiency renders UPS/MFS cells dependent on Skp2 which survives sarcoma cells by degrading p21 and p27; p16 is an important regulator in cell cycle through interaction with cyclin-dependent kinases (CDK).

The genetic alterations, such as mutation, deletion, epigenetic modifications, may be important for UPS/MFS development and progression, although non-specific. Further investigation had classified TP53, ATRX, H3F3A, ZFHX3, CSMD3, PRPRT, TRIO, CLTC, PDGFRB, ALK, PTCH1, RET, ERBB4, JAK3, GATA1, PIK3CG, RARA and MYH9 as “cancer driver genes” (Ali et al., 2019; Cui et al., 2022). Consistent with this finding, inactivation of tumor suppressor gene had been frequently reported in UPS/MFS. In some cases with TP53 mutation, p53 over-expression was detected (Cui et al., 2022). It is generally believed that p53 over-expression is associated with the recurrence, metastasis and poor prognosis. But the absent expression of TP53 was correlated with the positive expression of Skp2, driving cell proliferation by degrading p21 and p27 (Li et al., 2020) (Figure 1). Homozygous deletion of p16^INK4a^ was also observed and considered to be involved in the development of UPS/MFS (Simons et al., 2000; Hakozaki et al., 2006). Despite the detection of hypermethylation of p16^INK4a^ in some cases with loss at the region pinpointing CDKN2A (p16), there was no survival difference depending on the p16 expression (Niini et al., 2011) (Figure 1). It seemed that p16 might not be predominant senescence barrier in subtype of UPS/MFS, and the role of methylated modifications was unknown. However, the epigenetic screening found that methylation around the elements of ITGA10 and PPP2R2B, both of which were the upstream regulators of Akt/mTOR signaling. ITGA10 also impacted the downstream expression of TRIO and RICTOR, which activated RAC/PAK and Akt/mTOR signaling for UPS/MFS cell survival (Okada et al., 2016). Therefore, the epigenetic alterations around some signaling components might be involved in the sarcoma genesis and the progression of UPS/MFS.

**FIGURE 2:**
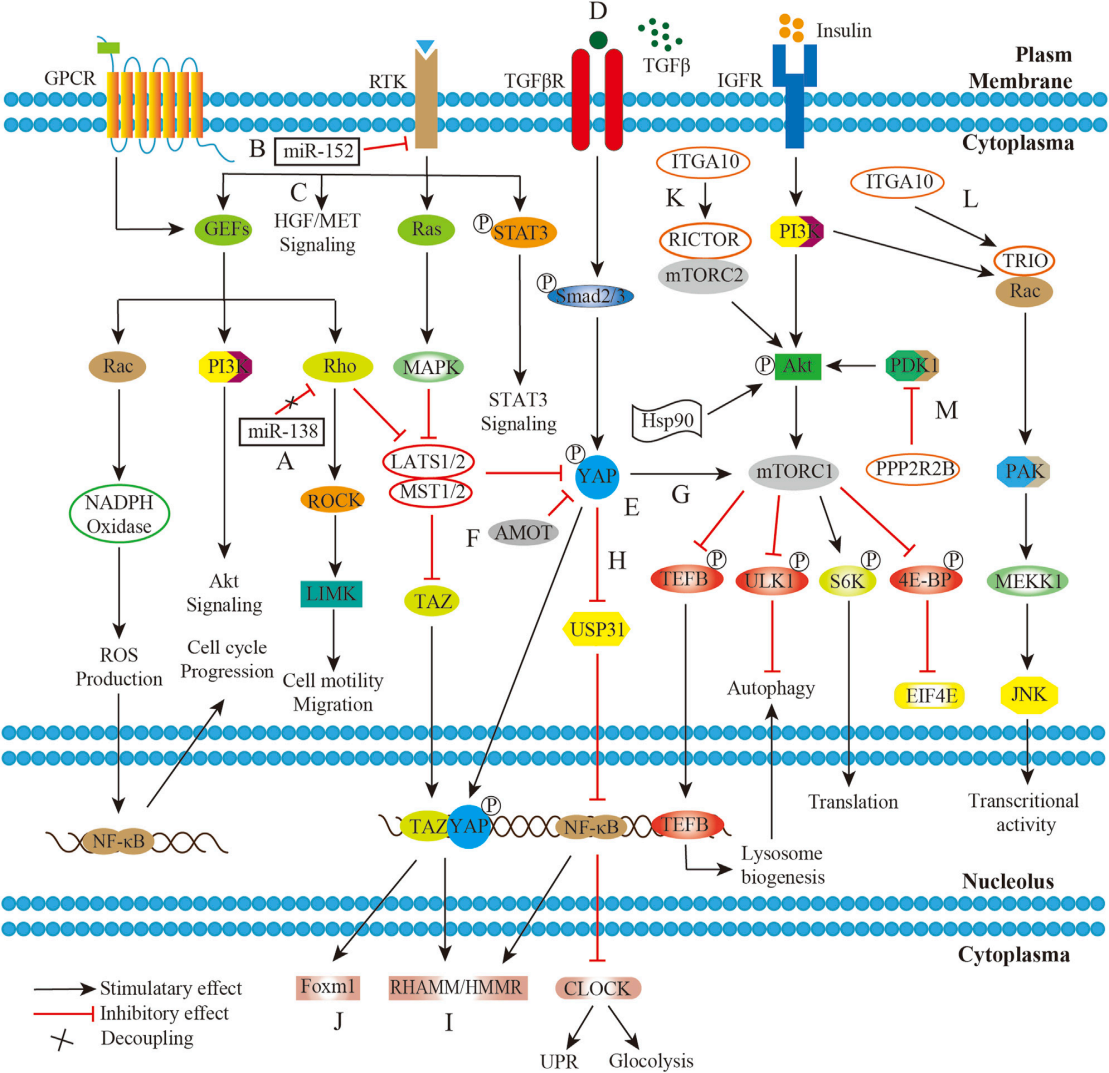
Working model of confluent network summarizing GPCR/Rho/ROCK, RTK/Ras/MAPK, TGFβ/YAP/NF-κB/mTOR, IGFR/PI3K/mTOR, and HGF/MET pathways in UPS/MFS. **(A)**. The decoupling of miR-138 from RHO-ROCK adhesion pathway promotes UPS cell migration; **(B)**. miR-152 downexpression disinhibits target genes production with receptor tyrosine kinase activity, and thus upregulates the downstream MAPK signaling; **(C)**. Hepatocyte growth factor/MET (HGF/MET) pathway was aberrantly activated due to its receptor overexpression. **(D)**. The significant secretion of TGFβ cytokine by tumor-infiltrating macrophages (TAMs) in the sarcoma microenvironment activates downstream signaling; **(E)**. Yes-associated protein (YAP) is constitutively activated by upstream pathways including TGFβ pathway; **(F)**. TAZ and YAP are normally inhibited by Hippo pathway or Angiomotin (AMOT), but unusually stable in UPS/MFS; **(G)**. YAP activates mTOR signaling, exhibiting NF-κB independent effect of on autophagy; **(H)**. YAP controls the expression of ubiquitin specific protein 31 (USP31), and thus phosphorylated NF-κB persistently suppresses the circadian clock activity, leading to cellular metabolism shift and unfold protein response (UPR) dysfunction; **(I)**. Stabilized YAP and TGFβ signaling cooperatively regulate hyaluronan-mediated motility receptor (RHAMM/HMMR) expression, enhancing sarcomagenesis and distant metastasis; **(J)**. The complex between transcriptional co-activator with PDZ-binding motif (TAZ) and YAP translocate into the nucleus and upregulate FOXM1 expression, which is pro-growth factor in UPS/MFS; **(K)**. The transcriptional product of ITAG10 is associated with RICTOR which is subunit of rapamycin complex 2 (mTORC2); **(L)**. ITAG10 encodes TRIO, and promote cell survival *via* RAC/PAK signaling; **(M)**. PPP2R2B encoding product directly interacts with PDK1 and suppresses AKT/mTOR signaling in UPS/MFS.

Aberrant activation of pathways in the UPS/MFS might be also driven by the component independent mechanism like receptor overexpression, altered transcription or post translational modifications. MET was a transmembranous tyrosine kinase receptor (TKR) in hepatocyte growth factor/MET (HGF/MET) pathway, and was well known as its critical role in pathogenesis of tumor. The MET gene amplification was identified in subset of UPS by immunochemistry and its receptor overexpression was detected by fluorescence *in situ* hybridization (Schmitz et al., 2015). Pathologically the activation of HGF/MET axis promoted cell proliferation and invasion. And the expression of both epidermal growth factor receptor (EGFR) mRNA and protein in UPS had been reported previously (Yamamoto et al., 2004; Hakozaki et al., 2006). However, it should be noted that the gene amplification was not always positively associated with the protein expression. The poor correlation between gene amplification and protein expression might be possibly due to the specific post-transcription processing. The observation that microRNA −152 (miR-152) downregulation led to an upregulation in TKR mRNA and protein levels in the UPS, and the dysregulation of TKR pathways were considered to play a role in sarcomagenesis (Pazzaglia et al., 2017). It was also reported that independent Rho/ROCK signaling members post-transcriptionally modified by miR-138 rendered primary UPS prone to be metastatic on the ground of the specifically over-expression of miR-138 in metastasis (Wong et al., 2015). In fact, the modified signal proteins with critical role also affect the biology and pathogenesis. Phosphorylation of Akt pathway components by overexpressed heat shock protein 90 (HSP90) significantly increased cell invasiveness and viability in specific subsets of UPS (Bekki et al., 2015). Independent of the activated Akt/mTOR signaling, phosphorylated STAT3 was downregulated in the JAK-STAT pathway, which was found to be associated with poor prognosis (Bekki et al., 2017). It, thus, will be necessary to determine the details of the molecular mechanism at different levels in this disease group (Figure 2).

However, the signal paths were organized non-linearly. They could form a network through a series of molecular interactions. The external signals could be transmitted, amplified and enhanced by the common extracellular receptor of multiple pathways. Insulin-like growth factor 1 receptor (IGF1R) was identified as the common upstream regulator of PI3K/mTOR and RAS/mitogen-activated protein kinase (MAPK) signaling, indicating its compensatory pathway activation after single pathway inhibition. Actually, both RAS/MAPK and PI3K/mTOR pathways were found to be activated in majority of UPS without oncogenic mutations (Serrano et al., 2016). Presumably, growth factors and its receptors were responsible for the hyperactivations of the pathways. And it was reported that co-inhibition of/PI3K/mTOR signaling and IGF1R could significantly reduce the cell growth, migration and invasion in the UPS (May et al., 2017). Similarly, the common signal transducer within multiple pathways held promise for a job on the co-activation. YAP1, together with TAZ (WWTR1), was generally inhibited in the Hippo pathway, but they were frequently activated in UPS. It was also linked to TGF-β signaling, which enhanced cell migration and invasion mediated by hyaluronan-mediated motility receptor (HMMR/RHAMM) (Ye et al., 2020). In the muscle-derived UPS, YAP1 suppressed unfolded protein response target genes and circadian genes. The effect on autophagy, metabolic disruption and hyper-proliferation was enhanced *via* Hippo/NF-κB axis (Rivera-Reyes et al., 2018; Ye et al., 2018). (Figure 2)

**FIGURE 3:**
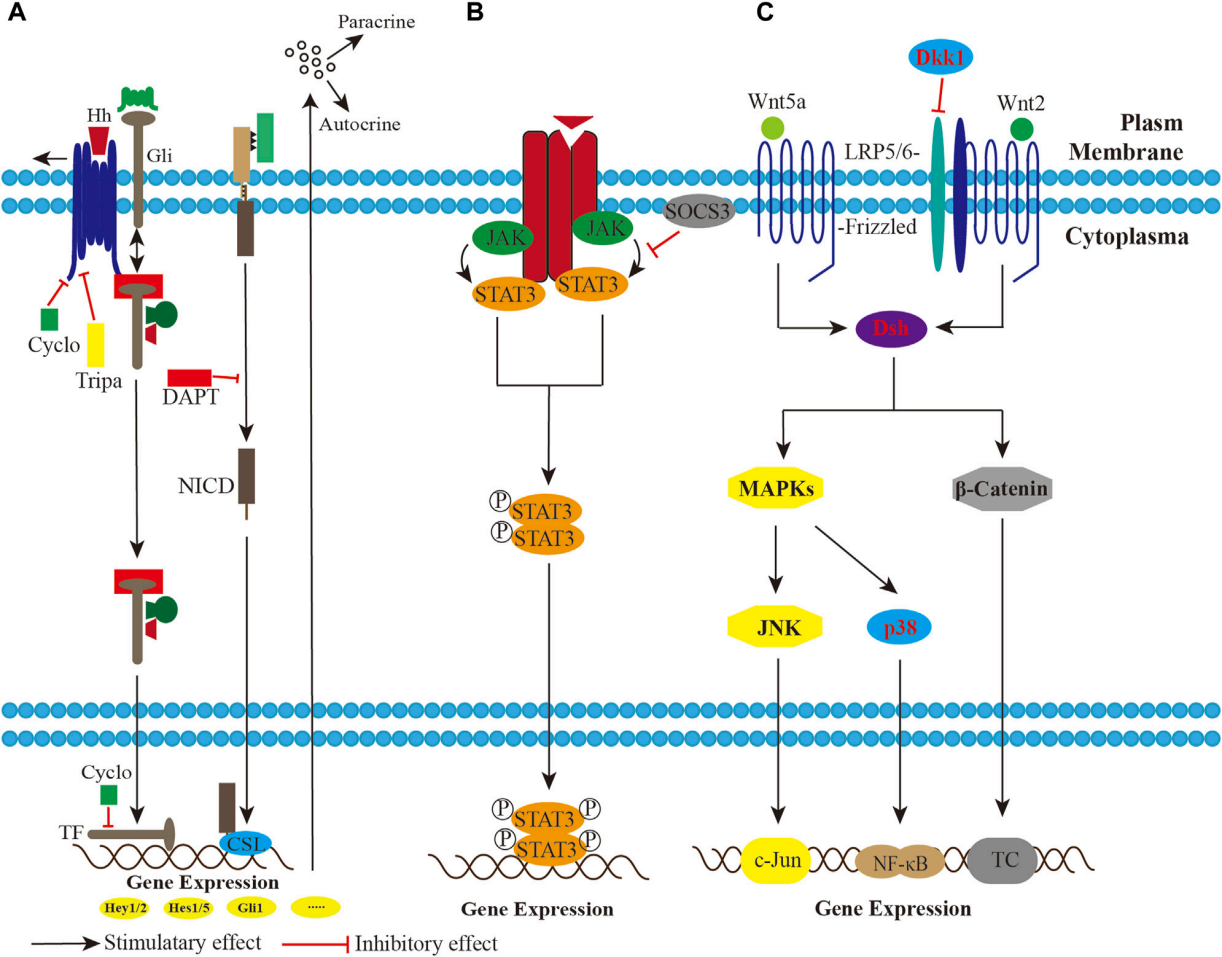
A schematic imaging showing the potential involvement of pathways in the UPS/MFS. **(A)**. The Hedgehog (Hh) and Notch signaling modulation are activated, and impose paracrine and autocrine effects on the sarcoma cells, maintaining mesenchymal stromal or stem cells in a less differentiated state. Pharmocological blockade by triparanol, cyclopamine and DAPT successfully inhibits Hh or Notch signaling. **(B)**. Phosphorylated STAT3 dimer functions as transcriptional complex (TC) in the Janus kinase (JAK)-signal transducer and activator of transcription (STAT) pathway. Suppressor of cytokine signaling 3 (SOCS3) overexpression has an effect on STAT3 dephosphorylation. **(C)**. Both Wnt2/β-catenin and Wnt5a/JNK non-canonical signaling inactivation inhibit developmental program in UPS/MFS. Wnt5a/JNK non-canonical signaling might be the upstream regulator of NF-κB signaling.

Similar to the heterogeneous tumors, a small fraction of cells with sarcoma-initiating potential rendered UPS/MFS self-renewal. In some cases, Hedgehog and Notch signaling were aberrantly activated due to the upregulation of the effectors, making stem-like cells in a less undifferentiated state (Wang et al., 2012). The inactivation of Wnt signaling also proved to be involved in the differentiation from human mesenchymal stromal or stem cells. Dkk1, the specific secreted protein of the Wnt developmental program, commonly exerted an inhibitory effect *via* Wnt2/β-catenin signaling. Conversely, reestablishment of both Wnt2/β-catenin and Wnt5a/JNK non-canonical signaling could reverse the poor differentiation (Matushansky et al., 2007). (Figure 3)

Meanwhile, some researchers focused on the resident chloride channels. Chronic chloride intracellular channels (CLICs) overexpression could lead to tumorigenesis. Previous studies suggested that IAA-94 affected cell growth and survival by inhibiting CLIC1, which was found to be widely expressed in the UPS (Murray et al., 2014). But the function of the CLIC1 pro-oncogenic pathway remains to be further elucidated.

### Presence of chromosome anomalies

Numerical and structural variants underlying chromosomal aberrations are frequently observed in the UPS/MFS, indicating the critical role of high chromosomal instability in the sarcoma genesis and progression. On one hand, the complex karyotypes with numerous marker chromosomes displayed aneuploidy, as indicated by the presence of multiple copies of chromosomes (Chr) and missing chromosomes. The events that each chromosome loss or gain in the analyzed metaphases, be they monosomy, trisomy or polysomy, were observed in respective investigation (Sreekantaiah et al., 1992; Aspberg et al., 1995; Schmidt et al., 1998a; Schmidt et al., 1998b; Mairal et al., 2000; Nishio et al., 2003; Nishio et al., 2010; Mertens et al., 2011; Becerikli et al., 2014). Of them, the most noticeable anomaly was trisomy, with a non-negligible percentage of pentasomy and hexasomy in comparison to that in normal human fibroblasts. But what calls for attention was that karyotype, which were identified as disomic group or tetraploidy, might be pseudodiploid or pseudotetraploid. According to the these investigations, Chr 2, 11, 12, 14, 18, 21, 22, Y frequently occurred loss, and the number for increase were for Chr 1, 3, 4, 6, 7, 8, 16, 17, 20. Furthermore a marked chromosomal aneuploidy varied from cell to cell within homologous clones. An observation that each chromosome presenting in 5–10 copies in >4% of the nuclei indicated the significant heterogeneity across the cell mass (Becerikli et al., 2014). Combined cytogenetic findings revealed chromosome counts varied between 22 and 180, with variations in a range from a near-haploid to hypooctaploidy modal chromosome number in different populations of cells (Sreekantaiah et al., 1992; Aspberg et al., 1995; Schmidt et al., 1998a; Nishio et al., 2003; Nishio et al., 2010; Mertens et al., 2011). On the whole, the tendency of numerical variants towards an increase or loss remains unknown. Another conundrum is that the cryptic mechanisms by which chromosomal aneuploidy occur are not fully understood, although the findings that impaired synchronization between chromosome duplication and cytokinesis, and the mutated tumor suppressor genes in UPS/MFS uncover the pathogenesis of aneuploidy in a small proportion of patients.

On the other hand, a plethora of structural aberrations had produced complex karyotype, in the form of rearrangement, ring chromosomes, telomeric associations, and dicentric chromosomes. Furthermore, high-level gains (amplification), namely, double minute chromosomes, homogenously staining regions (hsr), as well as add (19p) have been reported in UPS/MFS (Szymanska et al., 1995; Choong et al., 1996). But the majority of these structural changes detected by conventional cytogenetic analysis were non-specific for UPS/MFS, only a few were possibly not accidental. In general, chromosomal rearrangement was a recurrent structural variant, and the number of genetic gain and loss varied in a wide range lacking specific pattern, with a similar story told of the regions involved.

Firstly, with breakpoints analysis, numerous translocations were detected in the derivate or maker chromosomes as follow: t (1; 2), t (1; 3), t (1; 7), t (1; 10), t (1; 17), t (2; 3), t (5; 10), t (5; 11), t (5; 17), t (6; 8), t (6; 10), t (7; 10), t (9; 10), t (10; 11), t (10; 12), t (11; 17), and t (15; 21) (Mairal et al., 2000). And the fusion genes with potential involvement in sarcomagenesis, like TMTC-NTRK3, LMNA-NTRK1, were identified (Ali et al., 2019; Bai et al., 2022). Therefore, the chromosomal rearrangement was at play in the formation of composite karyotype.

Secondly, the reported number of copy number variation (CNV) range was from 2 to 168 (Mihic-Probst et al., 2004; Niini et al., 2011). However, the actual range of variant might be greater, due to the small number of cases in these researches.

Thirdly, the chromosomal regions with DNA CNVs were extensive and scattered. The array comparative genomic hybridization and the fluorescence *in situ* hybridization analyses showed that the most conspicuous copy number gains were for 1p12→p34.3, 1p21.3, 1p31.3→p31.2, 1p31→p32, 1p33→p32.3, 1p36, 1q11,1q21.2→q21.3, 1q21→q23, 2p21, 2q11.2→q21, 3p, 4p, 5p15.3, 6q11→q14, 6q22→qter, 7p12→pter, 7q22→q31,7cen→q11.2, 8p11.2, 8q12→qter, 8q24.21, 8q11.2→q21.1, 9q21→qter, 11p11, 11q13, 12q24, 14q11.2, 15q26, 15q21→qter, 16p13, 17p11.2, 19p13, 19q, 20q11.22, 20q13.2, and X; the regions with the most losses were at 1q41, 1q43→qter, 2q36.3→q37.2, 4q32→qter, 5q14→q23, 7q32→qter, 8p21→pter, 8q23, 9p21→pter, 9q31→q33, 10p11.2→p13, 10q11.2→q22, 10q25.3→q26.11, 13q, 13q13.3, 13q13.3→q14.11, 13q13.3→q14.2, 13q14.11, 13q14.11→q14.2, 13q14.3→q21.1, 13q14→qter, 16q12.1→q12.2, and 18q12→q22 (Tarkkanen et al., 1990; Mairal et al., 2000; Mihic-Probst et al., 2004; Kresse et al., 2010; Nishio et al., 2010; Niini et al., 2011). Gene amplifications were observed for 1p36, 1p32, 1q21→q23, 1q32, 3q26, 6q23, 4q, 5p, 7q, 8q21.2→q22, 8p23.1, 8q24, 9q31→q34, 10q26, 11q, 12q13→q15, 12p, 17q12, 20q (Tarkkanen et al., 1990; Sakabe et al., 1999; Mairal et al., 2000; Nishio et al., 2003; Mihic-Probst et al., 2004; Idbaih et al., 2005; Kresse et al., 2010; Nishio et al., 2010). Therefore, candidate genes in these loci, such as RB1, TP53, C-MYC, MDM2, ERBB, and KIT, are amplified or deleted, and the subsequent changes may be the first events in the sarcomagenesis and progression of UPS/MFS.

### Presence of protumoral microenvironment

The immune cells in the tumor microenvironment are found to play a protumoral role in UPS/MFS. Tumor-associated macrophages (TAMs) are known to produce significant cytokines, including TGFβ, and IL6, which could aberrantly activate downstream signaling and thus induce cell proliferation, migration, and invasion in UPS/MFS (Shiraishi et al., 2018; Ye et al., 2020). The percentage of TAMs was a prognostic factor for UPS/MFS. As correlative analyses of the SARC208 trial showed, patients with increased percentage of TAMs expressing PD-L1 were more likely to respond to Pembrolizumab and had a better progression-free survival (PFS) (Keung et al., 2020). In addition, infiltration of dentritic cells and neutrophils corresponded to different prognostic indicators such as recurrence-free survival (RFS) and disease-specific survival (DSS) (Hu et al., 2020; Cancer Genome Atlas Research Network and Cancer Genome Atlas Research Network, 2017). These results indicated that UPS had an inflammatory microenvironment. The analysis found that UPS had a highly expression of antigen presentation genes and regulatory T-cell genes (Pollack et al., 2017). These genetic alterations might contribute to robust oligoclonal T-cell infiltration that upregulated PD-L1 and other inhibitory ligands subsequently. Thus, UPS/MFS may be dependent on the immunosuppression within the microenvironment for immune evasion.

Altogether, multiple driver factors have been identified in subtypes of UPS/MFS. Furthermore, the crosstalk between pathways also plays a role in sarcoma genesis, proliferation, invasion, migration, and self-renewal. But, when it comes to who opens the Pandora’s Box of UPS/MFS, more work is required to elucidate the underlying mechanism or further subgroup this set of sarcomas.

## Conventional management of UPS/MFS

It is acknowledged that surgery remains the mainstay of treatment for all patients with localized UPS/MFS. But the infiltrative growth pattern of UPS/MFS is a negative factor for prognosis after surgery. Welsch studied the negative association of infiltration patterns with local control and advised separate assessment of all tumor margins against residual infiltrative “tail” (Welsch et al., 2018). Wide or radical excision involving the “tail” is required; otherwise, these sarcomas are prone to local recurrence and even metastasis. Wide excision followed by radiotherapy is typically recommended for deep lesions, but additional radiation might be limited for post-radiation UPS/MFS. Furthermore, inadequate removal of UPS/MFS may be hardly salvaged by postoperative radiotherapy (Greto et al., 2019), indicating that a negative margin significantly impacts local control and overall survival (OS).

Nevertheless, total removal of infiltrative sarcoma is a complex procedure constrained by inadvertent positive margins (IPMs) after UPS/MFS resection. Qualitative confirmation of IPMs is helpful in improving prognosis. However, preoperative radiation-induced fibrosis and previous surgery might cause margin alteration. Next, even when surgeons have been aware of these factors, they may still encounter an unsettled disputation of adequate margins for the mass resection. The adequacy of a negative margin from sarcoma is various. Kainhofer reported that local control rates were superior after R0 resection by Union for International Cancer Control -classification (minimal resection margin >1 mm) compared to R-classification (resection margin clear but allowing <1 mm) (Kainhofer et al., 2016). At Stanford University, researchers distinguished patients with a negative margin after R1 resection (1–4 mm) from those after R0 resection (>4 mm) in terms of distant metastasis rate (Kamat et al., 2019). But a retrospective study advocated a minimum resection margin of 10 mm for UPS/MFS and emphasized the significance of wide excision with ≥10 mm margin in local control compared to adjuvant radiotherapy (Fujiwara et al., 2020). Even a prospective study introduced a microscopic margin of ≥2.5 cm with a relatively acceptable 5-year local rate of 90% (Sampo et al., 2008). Sometimes, the use of wide excision may be limited in practice. Mohs micrographic surgery (MMS) likely offers better margin control and less tissue removal, thanks to the marked cytomorphology (McCoppin et al., 2012). It seems that the clinical outcome is superior to that after wide resection. Further investigations are still needed because the margin quality and dimension and the availability of margin data from all patients limit these results.

In fact, the oncologists seem to prefer wide excision for spinal lesions. A retrospective study identified subtotal or piecemeal total resection as an independent factor associated with OS and advocated a margin width of 2–3 cm (Lou et al., 2019). In particular, some clinicians championed *en bloc* vertebrectomy for those implicated in vertebrae. For those deep lesions involving major vessels or vital organs, systemic chemotherapy and/or palliative radiotherapy have gradually been accepted as appropriate.

In clinical practice, the choice of chemotherapy, the dose/cycle of treatment, the use of single or combined agents, and the latent toxicity have sparked numerous discussions on the care patterns, whereas most conclusions on the clinical decision-making process for UPS/MFS are heterogeneous.

In general, neoadjuvant/adjuvant chemotherapy improved OS in subsets of UPS/MFS, with first-line treatment being anthracyclines plus ifosfamide (A + I), well recognized as the most utilized regimen. However, the clinical response is limited and varied, which might be ascribed to the nature of high heterogeneity. Young et al. (2017) found an improved response to combined agents of doxorubicin-ifosfamide compared to doxorubicin alone (42.5% versus 6.9%) and better OS after combination chemotherapy in subsets of UPS. Clinicians also conducted randomization trials of Gemcitabine plus docetaxel (G + D) for UPS, which showed that G + D is not superior to A + I (Gronchi et al., 2020). But combination chemotherapy and the rising response rate at the expense of toxicities might be appropriate for young and fit patients. The clinical experience also validated that doublet chemotherapy was commonly seen in younger populations with advanced UPS (Bae et al., 2016). Nevertheless, whatever A + I or G + D, limited response to combination therapy has yielded an unsatisfactory prognosis for UPS/MFS (Hensley et al., 2002; Gronchi et al., 2012).

Accordingly, many efforts have been undertaken to develop novel therapeutic agent. Trabectedin is one of the hot-button drugs which has shown cytotoxic activity in UPS/MFS. Better still, Trabectedin might be the alternative option or subsequent therapy after A + I failure for UPS/MFS (De Sanctis et al., 2015; Martinez-Cruzado et al., 2017). However, trabectedin for UPS/MFS demands further efficacy and safety evaluation.

Another debate on the timing and cycle of chemotherapy in UPS/MFS is among clinicians. A Japanese trial (JCOG0304) on perioperative chemotherapy found limited patient benefits because of significant hematological toxicities (Tanaka et al., 2015). While some multicenter randomized clinical trials with long-term follow-up compared preoperative three and perioperative five cycles of epirubicin-ifosfamide. The outcome was comparable between these regimens, and it seemed that adjuvant chemotherapy, if needed, might be well administered preoperatively and limited to three cycles (Gronchi et al., 2016). However, this conclusion is not of universality due to its research design and the number of cycles.

## Targeting therapy

### Targeting druggable genes

Small wonder that identifying active targets for therapeutic interventions and establishing a novel signature to predict response to therapy are critical to management and clinical outcomes. The genomic and transcriptomic characterization of UPS identified TP53, ATRX, DOT1L, GCGR, COL4A2, KCNQ3, PKLR, SLC12A1, RARA, ALK, PTCH1, RET, ROS1, ABL1, MET, STK24(FARP1-STK24), ADAM17 (ASAP2-ADAM17), MMP20(PKNOX2-MMP20), NTRK1 (LMNA-NTRK1) as possible actionable genes, suggesting the potential use of immunotherapy. In fact, immunotherapy has gradually become a pillar in the powerful arsenal against advanced UPS. That dual agent checkpoint blockade immunotherapy plus radiotherapy can offer a complete response to patients with metastatic UPS is an inspiring news (Guram et al., 2018). Simultaneously, several immune therapy and radiotherapy investigations are underway to confirm the clinical utility (NCT03307616, NCT03116529, NCT03092323).

### Targeting tyrosine kinases

Tyrosine kinase receptors have been identified as the therapeutic target due to their specific molecular dysregulation in UPS. A Korean retrospective trial on Pazopanib for patients pretreated with cytotoxic chemotherapy showed a higher disease control rate in UPS group (Oh et al., 2020). But the efficacy of Pazopanib seemed unfavorable after the failure of the first-line treatment with doxorubicin or ifosfamide for advanced UPS (Kim et al., 2019). A real-world experience with Pazopanib in patients with UPS in Northern California showed that the PFS was approximately 3 months, and over 60% of patients with UPS developed progressive disease (Seto et al., 2019), which is significantly different from the result reported by the PALETTE trial (PFS: 4.6 months) (van der Graaf et al., 2012). But a retrospective study in Korea showed that patients with UPS pretreated heavily before Pazopanib also achieved acceptable clinical outcomes, with a mean PFS of 7.1 months (Yoo et al., 2015). Another Japanese study showed that the median PFS was 15.3 weeks, and the median survival was 9.5 months after Pazopanib treatment in the UPS group (Nakamura et al., 2016). Therefore, the efficacy and safety of Pazopanib vary with different settings. The number of patients, the previous lines of therapy, physical performance, the heterogenetic nature of UPS, and the race are the possible confounding factors affecting the outcome.

Anlotinib is another novel tyrosine kinase inhibitor targeting multiple factors involving VEGF/VEGFR signaling and fibroblast growth factor receptor. The PFS rate at 12 weeks was 58% for refractory UPS, and the median PFS and OS were 4.1 months and 11 months, respectively (Chi et al., 2018). Although the efficacy and safety were acceptable to some extent, the small number of patients with UPS in this trial demands multicenter clinical trials.

### Targeting anti-angiogenesis

Disruption of the tumor’s vasculature and interference with the vascular formation promises therapeutic strategies.

Endosialin (CD248), as detected by immunohistochemistry in most neoplastic cells of UPS and stromal fibroblasts, has been reported (Thway et al., 2016). Commonly, the expression of endosialin is significantly lost in the adult tissue. The sarcoma side population with renewal capability maintains the endosialin expression, and a randomized phase II trial of ontuxizumab (a humanized monoclonal antibody targeting endosialin) is underway (Sun et al., 2015). Consequently, the efficacy of this antibody remains to be evaluated.

Aminopeptidase N (CD13) expression is detected in higher-density UPS/MFS tumor cells. Further investigation into the prognostic impact of CD13 expression showed a significant association with relapse-free survival and OS. Torsten and his colleagues constructed the fusion proteins carrying NGR (asparagine-glycine-arginine) –containing peptides at the C-terminus of truncated tissue factor (tTF) binding to CD13 to inhibit sarcoma growth through vascular thrombosis. Although the therapeutic investigation of tTF-NGR against CD13 was performed in the human fibrosarcoma cell line, the prothrombogenic effect *in vivo* of these fusion proteins was independent of tumor histology (Kessler et al., 2018). These results suggest that it may be possible to use tTF-NGR for the treatment of UPS/MFS.

### Targeting tumor microenvironment

Hypoxia-induced factor (HIF) is critical in the hypoxic microenvironment, which may induce tumor cell migration. Elevated expression of HIF-1α is a strong predictor of UPS with metastatic potential. The mechanism might be that HIF-1α enhances the expression of the intracellular enzyme procollagen-lysine, 2-oxoglutarate 5-dioxygenase 2 (PLOD2), which promotes collagen modification and deposition. The subsequent study on the PLOD inhibitor minoxidil showed that HIF-1α–dependent induction of PLOD2 activity was required for cell migration (Eisinger-Mathason et al., 2013). PLOD2 appears to be a novel therapeutic target to reduce tumor cell dissemination.

Programmed death-1/programmed death-ligand 1(PD-1/PD-L1) inhibitor therapy may be a suitable treatment for UPS with a higher level of T-cell infiltration. Pembrolizumab, an anti-PD-L1 antibody, was assessed in a multicenter, phase II clinical trial (Tawbi et al., 2017). Meaningful clinical activity was observed in patients with UPS, and no devastating treatment-related adverse event was reported. But due to its heterogeneity, subgroups of UPS might not respond to PD-1/PD-L1 monotherapy. For example, macrophage with indoleamine 2,3-dioxygenase 1 (IDO1) pathway activation might conversely limit the efficacy of PD-1 because of the immunosuppressive tumor environment (Toulmonde et al., 2018).

### Targeting signal transduction

Signaling pathway members have been intensely scrutinized as potential therapeutic targets, promoting the development of inhibitors against the pathway’s components. The response of a genetically engineered mouse model closely resembling UPS to PI3K inhibitors BKM120 and BEZ235 seemed robust in delaying tumor growth. And the combination with doxorubicin significantly increased the complete response rate (Kim et al., 2012). Similarly, a dual PI3K/mTOR inhibitor, BGT226, combined with IGF1R inhibitor, AEW541, could synergistically reduce oncogenic activity and thus appeared to be a promising therapeutic strategy (May et al., 2017). These results showed that PI3K inhibitors were viable agents involved in the therapeutic regimen.

Neurotensin receptor 1 (NTSR1) was a component of GPCR, and its knockdown significantly prevented the aggressive behavior of UPS cells. SR48692, an inhibitor of NTSR1, was found to synergistically coordinate with chemotherapeutic agents to prevent UPS cell proliferation by inactivating extracellular kinase (Tokumoto et al., 2019). NTSR1 might be another target for UPS treatment.

Fibroblast Growth Factor 23 (FGF23), expressed explicitly in UPS of the bone compared to other sarcomas, was found to regulate cell proliferation, migration, and angiogenesis (Ali et al., 2019). FGF23 monoclonal antibody drugs with promising clinical results deserve further investigation in UPS.

### Targeting cell cycle

The regulation of gene expression by covalent modification of histones, transforming chromosome agglutination, or affecting the affinity of transcriptional elements, is a promising therapeutic agent for UPS/MFS. EPAS1, the gene encoding HIF-2α, was significantly silenced by epigenetic modification in UPS to adapt to the intratumoral hypoxia environment. The histone deacetylase inhibitor Vorinostat induced the re-expression of HIF-2α, thus suppressing tumor growth in an autochthonous model (Nakazawa et al., 2016). Caffeine can induce apoptosis, and valproic acid can act as a histone deacetylase inhibitor. It was found that their combination could synergistically produce a cytocidal effect in cell lines established from human UPS *via* cell cycle perturbation (Igarashi et al., 2017).

Eribulin, a novel synthetic agent targeting microtubules, inhibited microtubule dynamics and cell cycle arrest and finally initiated mitochondria-dependent apoptosis. A Japanese study reported that patients with UPS had higher OS with relatively tolerable adverse events (Nakamura et al., 2019). But larger-scale studies are required to evaluate the clinical outcome after Eribulin treatment.

In addition, the small-molecule agent YM155 selectively suppressed surviving, a poor prognostic biomarker known to inhibit mitochondrial apoptosis in a dose- and time-dependent manner in UPS/MFS cell lines (Minoda et al., 2015). It is suggested that activation of the mitochondria-dependent apoptotic pathway might be a therapeutic target.

## Potential therapeutics

Tumor-targeting *Salmonella typhimurium* A1-R, a facultative anaerobe that is an auxotroph of leucine and arginine, has amazingly shown strong efficacy *in vivo* and *in vitro* on patient-derived orthotopic xenograft (PDOX) models of UPS. Compared with the first-line therapy drug doxorubicin, A1-R was remarkably more effective against all PDOX models tested (Igarashi et al., 2018). These results suggest that bacterial therapy of S. typhimurium A1-R might be feasible for UPS.

In a murine xenograft model of UPS, Takeshi et al. provided a novel treatment with carbon dioxide (CO_2_). Transcutaneous CO_2_ application longer than 5 min could significantly decrease the tumor volume. Even CO_2_ treatment for ≥10 min could induce apoptosis (Ueha et al., 2017). In this regard, CO_2_ treatment might be useful and safe for further clinical trials.

In recent years, the success of adoptive cell therapy (ACT) and therapeutic cancer vaccine makes the cases for optimizing immunotherapeutic in UPS/MFS stronger. ACT including T-cell receptor (TCR) gene therapy, tumor-infiltrating lymphocyte (TIL) therapy, chimeric antigen receptor (CAR) T-cell therapy and natural killer (NK) cell therapy, is currently being investigated in sarcoma. There is limited clinical data available for UPS/MFS, albeit TCR therapy, and TIL therapy have been achieved objective response in other type of sarcoma. CAR T-cell therapy for UPS/MFS has emerged with the encouraging findings. Several UPS/MFS-associated receptors, EGFR, IGF1R, and TKRs, seemed to be amenable to this therapy (Pang et al., 2018). More mature results are awaited to prove the clinical efficiency of ACT in UPS/MFS, with some trials being under progress (NCT04052334, NCT03725605).

Cancer vaccine would be a promising therapeutic for UPS/MFS with greater immunogenicity. Delivery of antigen presenting cells into sarcoma could induce specific immune response with which there was a trend towards improved prognosis in the patients. Dendritic cells exposed to high Melanoma-associated antigen 3 (MAGE-A3) expression with poor prognostic indication, were detected in UPS/MFS. And the sufficient human leukocyte antigen expression and lymphocyte infiltration were beneficial for the antigen presentation in the UPS/MFS (Conley et al., 2019). Immunotherapy approaches targeting MAGE-A3 might have therapeutic value in the treatment of UPS/MFS. In fact, the cancer vaccine has been combined with chemotherapy or radiotherapy for soft tissue tumor including UPS in the previous clinical trials (Finkelstein et al., 2012; Krishnadas et al., 2015). But cancer vaccine still remains a highly charged issue.

## Summary and future perspectives

In general, UPS remains the diagnosis of exclusion. But with the identification of genetic/epigenetic alterations or chromosomal abnormities by comprehensive detection and the development of various treatments for UPS, the exact mechanism underlying the pathogenesis of UPS is sure to be clarified. In addition, whether differential investigations between UPS and MFS in future can provide new insights into their distinction also deserves more expectations.

## Abbreviations

A + I, anthracyclines plus ifosfamide; ACT, Adoptive cell therapy; CAR, Chimeric antigen receptor; CLICs, chloride intracellular channels; DSS, Disease-specific survival; FGF23, Fibroblast Growth Factor 23; G + D, Gemcitabine plus docetaxel; HIF, Hypoxia-induced factor; HGF, hepatocyte growth factor; HSP90, Heat shock protein 90; HMMR/RHAMM, hyaluronan-mediated motility receptor; IDO1, indoleamine 2,3-dioxygenase 1; IPMs, inadvertent positive margins; MACs, Tumor-associated macrophages; MAGE-A3, Melanoma-associated antigen 3; MFS, Myxofibrosarcoma; NTSR1, Neurotensin receptor 1; OS, Overall survival; PFS, Progression-free Survival; PLOD2, procollagen-lysine, 2-oxoglutarate 5-dioxygenase 2; PD-1/PD-L1, programmed death-1/programmed death-ligand 1; PDOX, patient-derived orthotopic xenograft; RFS, Recurrence-free survival; TCR, T-cell receptor; TIL, Tumor-infiltrating lymphocyte; UPS, Undifferentiated pleomorphic sarcoma.
